# Breast Self-Examination System Using Multifaceted Trustworthiness: Observational Study

**DOI:** 10.2196/21584

**Published:** 2020-09-23

**Authors:** Rajes Khana, Manmeet Mahinderjit Singh, Faten Damanhoori, Norlia Mustaffa

**Affiliations:** 1 Universiti Sains Malaysia Penang Malaysia

**Keywords:** trust, trustworthiness, multifaceted trust, breast self-examination, breast cancer, health care system, social media

## Abstract

**Background:**

Breast cancer is the leading cause of mortality among women worldwide. However, female patients often feel reluctant and embarrassed about meeting physicians in person to discuss their intimate body parts, and prefer to use social media for such interactions. Indeed, the number of patients and physicians interacting and seeking information related to breast cancer on social media has been growing. However, a physician may behave inappropriately on social media by sharing a patient’s personal medical data excessively with colleagues or the public. Such an act would reduce the physician’s trustworthiness from the patient’s perspective. The multifaceted trust model is currently most commonly used for investigating social media interactions, which facilitates its enhanced adoption in the context of breast self-examination. The characteristics of the multifaceted trust model go beyond being personalized, context-dependent, and transitive. This model is more user-centric, which allows any user to evaluate the interaction process. Thus, in this study, we explored and evaluated use of the multifaceted trust model for breast self-examination as a more suitable trust model for patient-physician social media interactions in breast cancer screening.

**Objective:**

The objectives of this study were: (1) to identify the trustworthiness indicators that are suitable for a breast self-examination system, (2) design and propose a breast self-examination system, and (3) evaluate the multifaceted trustworthiness interaction between patients and physicians.

**Methods:**

We used a qualitative study design based on open-ended interviews with 32 participants (16 outpatients and 16 physicians). The interview started with an introduction to the research objective and an explanation of the steps on how to use the proposed breast self-examination system. The breast self-examination system was then evaluated by asking the patient to rate their trustworthiness with the physician after the consultation. The evaluation was also based on monitoring the activity in the chat room (interactions between physicians and patients) during daily meetings, weekly meetings, and the articles posted by the physician in the forum.

**Results:**

Based on the interview sessions with 16 physicians and 16 patients on using the breast self-examination system, honesty had a strong positive correlation (r=0.91) with trustworthiness, followed by credibility (r=0.85), confidence (r=0.79), and faith (r=0.79). In addition, belief (r=0.75), competency (r=0.73), and reliability (r=0.73) were strongly correlated with trustworthiness, with the lowest correlation found for reputation (r=0.72). The correlation among trustworthiness indicators was significant (*P*<.001). Moreover, the trust level of a patient for a particular physician was found to increase after several interactions.

**Conclusions:**

Multifaceted trustworthiness has a significant impact on a breast self-examination system. Evaluation of trustworthiness indicators helps to ensure a trustworthy system and ethical interaction between a patient and physician. A new patient can obtain a consultation by referring to the best physician according to preference of other patients. Patients can also trust a physician based on another patient’s recommendation regarding the physician’s trust level. The correlation analysis further showed that the most preferred trustworthiness indicator is honesty.

## Introduction

### Background

Breast cancer has become the most prevalent type of cancer affecting women in Indonesia and worldwide, and the number of deaths caused by breast cancer is growing every year [1,2]. In 2018, there were an estimated 2,088,849 new cases and 626,679 mortalities related to breast cancer [1]. Breast cancer occurs with an increase in the number of malignant cells originating from the inside layer of the mammary glands [3]. In the United States, breast cancer is detected by mammography (43%, 156/361), breast self-examination (25%, 90/361), clinical screening with breast self-examination (14%, 47/361), and accidents (18%, 64/361) [4]. Breast cancer prevention requires every woman to perform a breast self-examination as an early diagnosis mechanism for all ages after the first menstruation, which is expected to help reduce breast cancer mortality [5-8].

Moreover, curiosity about seeking health care–related information through the internet and social media has been gradually on the rise. Social media users prefer to seek information from social media [9,10], as other users share a substantial amount of information pertaining to breast cancer. Almost 87% of the total posts on Facebook related to breast cancer consist of support groups [11]. Other platforms such as Twitter include surveys on breast cancer education, shared stories about breast cancer survival, treatment plans, and images showing the progress of certain treatments [12]. Consequently, patients prefer to use social media to talk about sensitive body issues (such as breast cancer) as a more convenient venue than face-to-face interaction with a physician [13-16]. At the same time, physicians are actively participating in social media and health care systems related to breast cancer [9,14,17,18], and they tend to use social media for assisting, treating, and consulting on cancer [9,18,19]. Although the physician-patient interaction on social media platforms offers many conveniences, it also has important downsides.

There are reported cases of physicians behaving inappropriately on social media, such as posting incorrect information, misrepresenting their credentials, posting improper content, and false advertising [20-22]. The impact of such unethical behavior [19,23-26] can result in embarrassing patients and losing their trust. Since trustworthiness is an essential factor in any physician-patient relationship, the decrement of trust not only affects the health care business but also causes shame and depression for the patient [20,21,23]. Thus, in this study, we explored a trust model that can support and eliminate this issue. Toward this end, we focused on enhancing the multifaceted trustworthiness model proposed by Quinn et al [27] to be adopted for health care treatment on social media. The current multifaceted trust model calculates a trust score based on social interaction on social media platforms. Thus, the two-way trust evaluation adopted in this trust model is suitable for considering the patient-physician interaction in the health care domain. However, the multifaceted model has its limitations, since there is no credential representation mechanism in building trust context, no informed consent contract between parties trying to build trust, no mechanism to protect confidential data [19], and no preservation of user privacy [14]. Thus, there is a gap to be filled regarding how to best protect confidential information and ensure that each interaction and communication on social media is based on ethics.

The trust in social media is not personalized, specific, and single-faceted, but is rather generalized in a group context [27-29], and trust level cannot be annotated [27,28]. By contrast, the existing multifaceted trust model is personalizable, specializable, and capable of measuring the accuracy of trust recommendation [28]. As a result, Chieng et al [30] presented personalized comments or photos on social media as a user-centric model.

The objectives of this study were to: (1) identify the trustworthiness indicators that are most suitable for a breast self-examination system, (2) design and propose a breast self-examination system, and (3) evaluate the multifaceted trustworthiness interaction between patients and physicians using the breast self-examination system. Implementation of the multifaceted trustworthiness model into the breast self-examination system can identify the most preferred indicator of trustworthiness, offering relationship feedback between the patient and physician based on trust value and trust level. This could ultimately provide a more trustworthy and ethical patient-physician interaction on social media platforms such as Facebook.

### Principles of Trust and the Multifaceted Trust Model

The trust theory introduced by Rotter [31] in 1967 is known as interpersonal trust, defined as “an expectancy held by an individual or a group that the word, promise, verbal or written statement of another individual or group can be relied upon.” The trust principle was introduced by Mayer and Davis in 1995 [32], which posits that factors related to the trustor and trustee will lead to trust. The characteristic of the trustor is trust propensity, which is a general willingness to trust others. In other words, trust propensity is a cause of risk behavior. People with different backgrounds, personality types, cultures, and experiences will differ in their propensity to trust.

On the other side, the main characteristic of the trustee is trustworthiness, which is measured as the motivation to lie. For example, if a trustee will gain something through lying, they will be seen as less trustworthy [32].

Trust can therefore be defined as the confidence in somebody or a belief that somebody is good and honest [33]. Trustworthiness has been mentioned in the context of character honesty and integrity in health care [34], and it is a context-dependent and personalized characteristic [30]. According to Quinn [28], the multifaceted trustworthiness model is personalizable and specializable [28]. The trust characteristic in social media has been defined according to the following four traits [30,35-37]. The first is asymmetry, as trust between two users is not identical. That is, individual A could trust individual B, whereas individual B might not essentially trust individual A and vice versa. Second, trust is transitive: longer links create less trust. For example, consider that A and B are friends, and they trust each other. B is friends with C, but A does not know C. Since A knows B and trusts B’s friends, A might trust C to a certain extent. However, C is friends with D, whom neither A nor B knows, and thus A finds it difficult to trust D because of the distant connection and the fact that they do not know each other. Third, trust is context-dependent, according to time, situation, and experience. People tend to exhibit differences in trust based on the context. Fourth, trust is personalized as a subjective view. That is, the trustworthiness of a particular person might be viewed differently by two different people.

**Figure 1:**
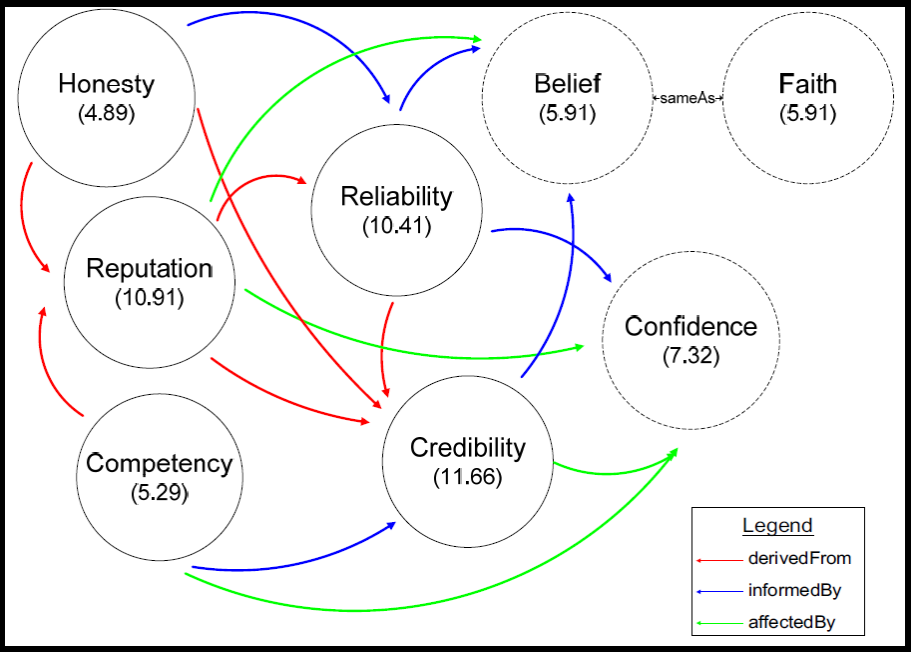
Personalized model of trustworthiness [27].

The key indicators of the multifaceted trust model (Figure 1) are honesty, reputation, competency, reliability, credibility, belief, confidence, and faith [27]. Honesty means the person makes good-faith agreements, tells the truth, and fulfills any promises made. Competency is the ability of one person to fulfill another person’s needs. Confidence is “a feeling of certainty or easiness regarding a belief one holds” [38]. Reputation is part of the social notion of trust [39]. Belief is justified and should be accepted (ie, acceptable without argumentative support) [34].

Figure 1 shows how the concept of personalization allows the user to declare the idea that competency is influenced by reputation (ie, competency derived from reputation), credibility is influenced by belief (ie, credibility informed by belief), and so on. This concept can be repeated to construct a trust model to suit the user’s needs and to reflect the user’s subjective view of trust.

### Trust Measurement Models

The trust measurement model in an open social network can be classified into five main models [36]: online reputation models, Marsh trust management, multicontext trust, trust inference for social networks (TISoN), and action-based trust.

#### Online Reputation Model

The online reputation model is based on online marketplaces such as Amazon and eBay. These models focus on user performance ratings provided after every part of the transaction is completed. The reputation value is then derived from the total sum of scores on eBay and the mean value from all ratings on Amazon. There would be no mechanism on correction if the user provided false information, as this model runs only based on the increment number of opinions that can create the reliability of reputation value [40].

#### Marsh Trust Model

Marsh [41] proposed a trust model based only on direct interactions, which can be broken down into basic trust, general trust, and situational trust.

For basic trust, the agent has an independent trusting disposition, which is calculated based on the accumulation of agent experiences. The best experiences bring an excellent disposition to trust, and the minimum experiences bring a bad disposition to trust. Marsh presented the notation *T_x_^t^* to identify the trust disposition of agent *x* at time *t*.

For general trust, the trust of the agent does not consider factors of the specific situation. Marsh used the notation *T_x_(y)^t^* for expressing general trust between agent *x* and agent *y* at time *t*.

For situational trust, the trust of agents takes into account the specific situation. The following formula is used to calculate situational trust based on the utility of a situation:


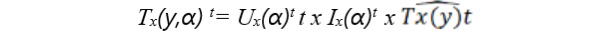


in which *x* is the evaluator, *y* is the target agent, and *α* is the situation. *U_x_(α)^t^ t* represents the utility *x* taken from situation *α*, *I_x_(α)^t^* is the important aspect in the situation *α* to agent *x,* and 
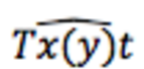
is the general trust estimation when identifying all possible data into *T_x_(y,α)*.

#### Multicontext Trust

Based on the Marsh trust model described above, a model in which context trust represent the fields of trust capability was proposed. For this purpose, trust is broken down into different contexts and each context is normalized in the range of 0 to 1 to fulfill future aggregation. The following seven trust functionalities on Facebook are considered [42]: (1) interaction time span (*S*), (2) number of interactions (*N*), (3) number of characters (*C*), (4) interaction regularity (*F*), (5) photo tagging (*P*), (6) group membership (*G*), and (7) common interests (*L*).

These seven contexts are summed to establish the formula of trust aggregation. Marsh [42] multiplied these contexts and used the final values, which identified a vector with several numbers where *T_x_* is the priority given context P=(T_S_, T_N_, T_C_, T_F_, T_P_, T_G_, T_L_), and the final value of trust is formulated as:


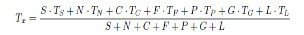


The method of aggregation is important for attributing a value for each context. As an example, the context-free contribution to the overall trust simply represents decreasing the level of importance to the priority vector [42].

#### TISoN

The computational model for TISoN was introduced as a hybrid model based on a mathematical model and algorithm. Hamdi et al [43] also generated and evaluated trust values for relative rating. The authors designed a novel trust path–searching algorithm to ensure reliability of the trust path in a wider social network and used the trust inference measure to measure the degree of user trust in others.

#### Action-Based Trust

Gambhir et al [44] introduced action-based trust as a new model of trust based on user content disclosures such as comments, “likes,” post sharing, image tagging, and video posting. The algorithm of action-based trust involves calculation of trust values for user actions performed that focus on sharing sensitive content in an online social network. This algorithm has also been used by the multifaceted trust model in the context of online social networks [44].

### Breast Self-Examination System in Online Social Networks

Breast self-examination is a method for early detection by which women examine their breasts to facilitate detection and alleviate any fear of cancer [45]. Breast self-examination is a regular monthly breast check using a mirror to observe any abnormal changes on the breasts [3], and is also considered to be the best tool for early breast cancer detection [46].

As patients prefer using social media to make appointments, receive reminders, diagnostic test results, provide information about their health, and as a forum for asking general questions related to health care [14], some specific features have been requested by patients as a reference to develop a breast self-examination system. Based on existing breast self-examination systems (Table 1), nine standard features should be embedded in any online breast self-examination system, including user account management, calendar, self-exam wizard, history, chat room, location, knowledge, video tutorial, and forum.

**Table 1:** Comparison of currently available breast self-examination (BSE) systems.

Existing BSE System	Features								
	User Account	Calendar (cycle period)	Self-Exam Wizard	History	Chat Room	Location	Knowledge	Video Tutorial	Forum
BSE Apps [47]	Absent	Present	Present	Present	Absent	Absent	Absent	Absent	Absent
Keep A Breast App by Luis M [48]	Absent	Present	Present	Absent	Absent	Absent	Absent	Absent	Absent
Beyond The Shock App by NBCF [49]	Present	Absent	Absent	Absent	Absent	Absent	Present	Present	Present
Dr K's Breast Checker App [50]	Absent	Absent	Present	Present	Absent	Present	Present	Absent	Absent
Daisy Wheel App [51]	Absent	Absent	Present	Absent	Absent	Absent	Absent	Present	Absent
Breast Control App [52]	Absent	Present	Present	Present	Absent	Absent	Present	Absent	Absent
Makna (LUDIc) [53]	Absent	Present	Present	Absent	Absent	Present	Present	Present	Absent

### Theory of Physician-Patient Interaction

The physician-patient interaction is a communication process that describes the shared nature of the problem, treatment aims, and psychosocial care [54]. The physician-patient interaction emphasizes the behaviors of physicians toward patients. Behavior consent is the content given by the physician on health solutions (instrumental behavior) and the capability of physicians to treat patients (affective behavior) [55].

## Methods

### Research Flow

**Figure 2:**
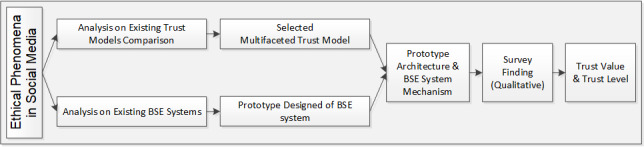
Research flow. BSE: breast self-examination system.

The research flow is outlined in Figure 2, following ethical phenomena in social media. The multifaceted trust model was selected for this study, as it is able to provide a subjective view of individual trust for each user. The prototype of the breast self-examination system was based on existing breast self-examination systems (Table 1). The architecture of the breast self-examination system with trustworthiness indicators was designed as a rating for the trust value for each patient consultation with a physician. Based on a survey with patients and physicians, the relationship between a particular physician trust value and trust level was identified. Thus, any patient who wants to choose a physician for consultation could refer to the physician’s trust level.

### Existing Breast Self-Examination Systems

The comparison among the existing breast self-examination systems in Table 1 highlights the user account as an essential feature, which is based on the Beyond the Shock app [49] user account for securing patient and physician data. The remaining six apps do not have user accounts because they do not secure patient data, especially with respect to female breasts. The calendar is the feature that establishes the menstrual cycle period for performing a monthly self-exam. Of the seven available apps, four (BSE, Keep A Breast, Breast Control, and Makna) provide a calendar for setting a monthly self-exam reminder. The self-exam wizard feature explains how to perform a self-exam systematically, which should exist in any breast self-examination system. Only Beyond the Shock does not provide this feature. The history feature helps to record the patient’s activity and the interaction process with their physician, which is present in several apps (BSE, Dr. K’s Breast Checker, and Breast Control). The history feature records data by keeping a medical history for each patient, which will help physicians to trace each patient’s performance. It is vital that this feature is secure due to the private nature of the information. The chat room allows for direct interaction between the patient and physician, which is essential for allowing patients to have direct interaction and communication with a physician without a face-to-face meeting. None of the apps currently supports the chat room feature, as they focus only on self-exam without connecting to a physician.

The next feature is location, which identifies available physicians nearest to the patient. This feature helps patients find a physician for further consultation based on their self-exam results. This feature is found in Dr. K’s Breast Checker and Makna*,* which provide an address of a hospital or clinic for consultation with a physician. The knowledge feature provides scientific information related to breast cancer prevention, which is also an essential feature for obtaining breast cancer–related information for patient education on breast cancer. This feature is offered by several apps (Beyond the Shock, Dr. K’s Breast Checker, Breast Control, and Makna)*.* The video tutorial feature refers to any related breast cancer information provided via video as guidance. Video tutorials are provided in Beyond the Shock, Daisy Wheel, and Makna as an essential feature to help patients view information related to living with breast cancer. The forum is an open space to find current news or cases from physicians and patients, which offers a space where physicians provide general information to all patients on breast cancer prevention. Beyond the Shock uses a forum as an essential space for discussion between all physicians and patients in the same area. We included all criteria shown in Table 1 in our proposed breast self-examination system.

### Integration of the Multifaceted Model of Trustworthiness in the Breast Self-Examination System

We refer to Quinn et al’s [27] multifaceted trust model, which uses the idea of implementing a trust management model to act as the subjective view on trust. The breast self-examination system involves the eight indicators of trustworthiness as a rating system in a chat room setting. These indicators will determine the value of trust based on the user’s interaction experience that is entirely personalized, transitive, and context-dependent. The personalized view will allow users to choose their trust value (ie, patients will give a value to each indicator in reference to their physician, and vice versa). The transitive view will offer each physician trust value as a reference when a patient recommends a physician to another patient. The context-dependent view will give patients flexibility in rating a physician; they can edit their trust level regarding physicians from time to time based on several consultations.

The trust level task is designed through the average rating value (ARV), which is used to calculate the average trust value given by the patient to their physician. The ARVs were generated based on the idea of Marsh [41] and Daskivich [56] to identify the trustworthiness level of a physician or patient with independent values. The trustworthiness level and independent value of a physician denoted by ARV are shown in Table 2.

**Table 2:** Trustworthiness level scale.

Trustworthiness level	Average rating value (ARV)	Independent value
High	9.0≤ARV≤10.0	5
Medium	7.5≤ARV<9.0	4
Low-Medium	5.0≤ARV<7.5	3
Low	2.5≤ARV<5.0	2
Distrust	0≤ARV< 2.5	1

If ARVs are between 9 and 10, the trustworthiness level is considered to be high, and the independent value is 5, whereas lower values indicate higher levels of distrust. Therefore, the correlation analysis will depend on the independent value as the critical element.

### Prototype Architecture and Mechanism of a Breast Self-Examination System

The prototype architecture of our breast self-examination system was based on Quinn et al’s [27] trust model, miniOSN [30], and miniOSN2.1 [30]. Patients can personalize accessibility to posted information, comments, and shared history data using the rating feature, as well as limit the physician to view the content of the patient data. The patient allows viewing the trust value and trust level of physicians. A physician’s trust value is based on the average of the trustworthiness indicators [28] and the ARV as the rating trust level [41,56]. The trust level of a physician is then identified by the trust value. To identify the ranking among multifaceted trustworthiness indicators, we evaluated the relationships between each indicator and the trust value. The higher ranking of a trustworthiness indicator is determined by a stronger correlation between the trust value and each indicator. Therefore, the ranking could evaluate the importance of the trustworthiness indicator in the multifaceted model. In the breast self-examination system, patients can personalize accessibility to set the value of posting and comments according to the trustworthiness indicators or trust level.

**Figure 3:**
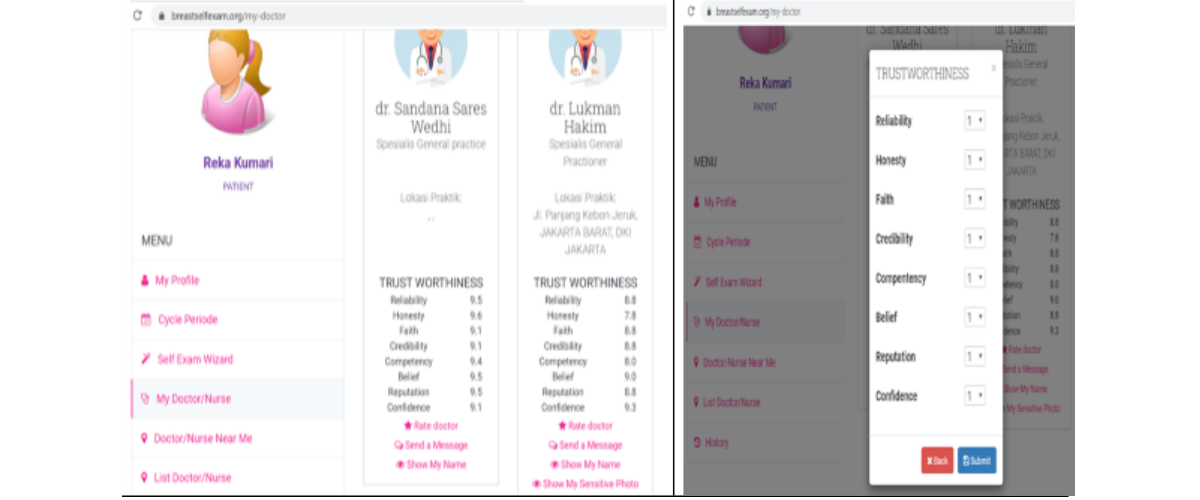
Left: Patient rated two physician after interaction; Right: Trustworthiness scale.

The trustworthiness mechanism on the breast self-examination system is measured through chat rooms and forums. When a patient request is accepted by a particular physician for consultation, the patient will encourage the trust level option of the physician, which is only seen by the patient. The patient can edit the trust level of a physician by choosing values ranging from 1 to 10 from the trustworthiness indicator’s ARV. The patient can also update the level of trust from time to time. For example, Figure 3 shows that the request of Reka (patient) for a consultation with physicians Sandana and Lukman was accepted by both physicians. After the meeting, she gave the experience a rating of 8 as the value for the physicians.

### Action-Based Trust Algorithm for the Breast Self-Examination System

An action-based trust algorithm was further implemented for the computational mechanism on the proposed breast self-examination system. This algorithm can measure the credibility of users on social media. The capabilities for evaluating and calculating the trust factors on user content disclosure include sharing personal records, sharing a post, comment, photo, and posting a message [44]. The computation of trust value is for a physician that acts on user content disclosure, namely as a trust factor. A physician trust factor may decrease or increase based on whether the patient selects a sensitive or not sensitive option. For example, the patient will likely select a sensitive option for a self-exam photo after completing the monthly self-examination. The sensitive option must be accompanied by informed consent from the patient before it is shared with the physician.

The action-based trust algorithm divides the measurement of each user action into weights, including the weight of action (Wa), weight of post (Wp), and weight of category (Wc). At the same time, Wc is a function of the weight for category. These weights, Wa, Wp, and Wc, are identified as the parameters of the trust factor. Table 3 shows the cluster of weights for test cases simulating the algorithm [44].

**Table 3:** Weighted clusters in the action-based trust algorithm.

Weight type		Weight value	
**Action**			
	Share		0.008
	Like		0.006
	Comment		0.007
	Dislike		0.006
	Tagging		0.005
	Post		0.008
**Post**			
	Photo		0.003
	Video		0.002
	Link		0.001
	Message		0.003
**Category**			
	Sensitive		0.009
	Nonsensitive		0.001

### Survey

We conducted a survey using open-ended interviews with 32 participants [57,58] and 77 interactions in the breast self-examination system. The survey was conducted from February 3, 2020 to March 30, 2020. The participants were physicians and female outpatients, all of whom had used the breast self-examination system. The 32 participants included 20 females and 12 males, comprising 16 physicians [58] and 16 female outpatients [57]. Of the 16 physicians, 12 were general practitioners and 4 were oncology specialists.

The 16 outpatients were healthy females who were aware of the health care system. Eight of these outpatients were aged 18 to 25 years old and the other eight were aged above 25 years. However, not all 32 participants ultimately completed the interaction task in the chat room and forum due to the consultation period. There were 24 active chat room participants and 22 participants interacted in the forum. The evaluation monitored the activity in the chat room (interaction between physician and patient) and the sharing of information by the physician through the forum. The interview started with an introduction to the research goal and an explanation of the flow on how to use the breast self-examination system.

This study was approved by the research ethics board of Esa Unggul University committee (No. 0155-20.133/ DPKE-KEP/ FINAL-EA/ UEU/ V/ 2020).

## Results

### Design of the Breast Self-Examination Prototype

**Figure 4:**
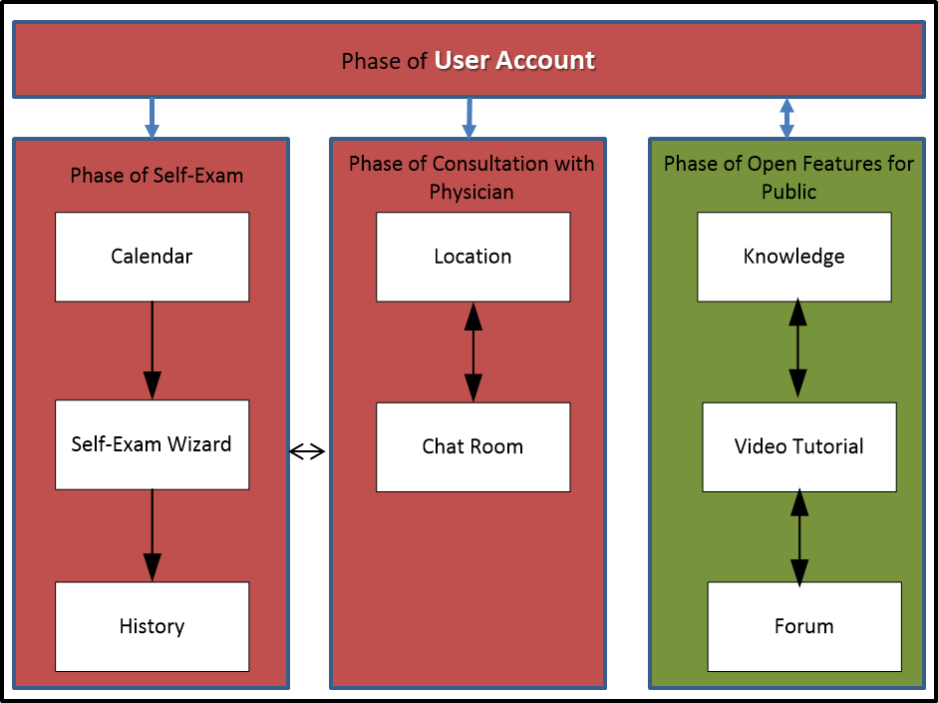
Module design of the breast self-examination (BSE) system [59].

The prototype follows a module design (Figure 4), which is classified into four phases [59]. The first phase of the user account is registering and logging into the system. The second phase of self-exam is the phase of conducting a personal self-exam on the breast and annotation into the system. The third phase of consultation with a physician is when the user finds a doctor and has a consultation. The fourth phase of open features for the public is the opportunity for the public to access knowledge, video tutorials, and forum features without obtaining a user account. The phrases in each phase are based on user privileges.

Each of the features of the breast self-exam system shown in Figure 4 has its own function. The calendar is a reminder system for performing a breast self-exam. The self-exam wizard is a guide to performing the correct self-exam daily. History serves as a self-exam record and tracks monthly breast self-exams. The location finds the nearest physician for receiving treatment. The chat room is a space for consultations between patients and physicians. Knowledge is a collection of links to news and expert opinions on breast health. Video tutorial is a collection of videos for performing the breast self-examination correctly. The forum is where physicians can share important information for patients related to breast cancer or breast self-exam.

### Selection of a Suitable Trust Model

To select the most appropriate trust model for the breast self-examination system, we performed a literature review on papers related to the trust model. Ultimately, 11 articles were selected for comparison analysis among existing trust models. Table 4 shows the analysis of the comparison from several perspectives, including the trust model, related domains, selection of trust factors, methodology, and benefits.

The selection of trust models to suit the breast self-examination system refers to the health care, internet, and social media domains. Among the 11 articles, 7 are related to health care, 2 pertain to the internet, 1 is associated with social media, and the other is related to psychology. The trust model related to social media is the Multifaceted Trust Model for Online Social Network Environment [30]. Initially, the multifaceted trust model was introduced by Quinn [27] for the internet environment. The multifaceted trust factors support a user-centric model that requires users to personalize trust. For instance, Abbas et al [60], Montaque et al [61], and Quinn et al [27] focus on different areas with respect to reliability. Abbas et al [60] focus on the overall reliability of health care software, and Montaque et al [61] focus on the overall reliability of medical technology. In contrast, Quinn et al [27] and Chieng et al [30] introduce reliability as being personalized and specialized to a particular user or things.

From the methodology perspective, 5 of the studies were based on a qualitative approach, 5 were based on a quantitative approach, and the remaining study was based on a structured literature review. The qualitative approaches include evaluation of trust in the relationship between the patient and physician [25,63,64], whereas the quantitative approaches concentrate on a questionnaire to obtain participants’ feedback. Therefore, the qualitative approach is more effective and relevant for garnering maximum performance out of the system.

The benefit perspective brings a consistent approach to choosing the trust model for the breast self-examination system. Two of the studies explored the trust model benefit that focused on the personalized and trust recommendation measurement offered by Quinn et al [27] and Chieng et al [30]. In contrast, the remaining trust model benefits focus on the general trust model of theoretical issues such as the instrument created [63], trust theory on the patient-physician relationship [25], patient trust in technology [61], and behavior approach theory [64]. Thus, a trust model related to the user-centric model is relevant to be embedded in the breast self-examination system.

**Table 4:** Comparison of existing trust models.

Reference	Trust Model	Domain	Trust Factors	Methodology	Benefit
Abbas et al [60]	Trustworthiness health care software model	Health care	Safety, validity, reliability, reusability, scalability, maintainability, performance	Structured literature review	The initial definition of trustworthiness attributes identified.
Velsen et al [63]	A conceptual model of patient trust in telemedicine services	Health care	Trust in the care organization, trust in the care professional, trust in the treatment, trust in the technology	Qualitative method on focus groups with a survey on four factors (trust in care organization, care professional, treatment, and technology)	A valid instrument (PATAT) created to assess patient trust in a telemedicine service and as a benchmark on the same service.
Krot and Rudawska [25]	Model of trust in the doctor-patient relationship	Health care	Macrotrust, microtrust, mesotrust	Qualitative method on the analysis of published comments	Trust in the doctor-patient relationship is a social, complex, and multidimensional phenomenon.
Chieng et al [30]	Multifaceted trust model for online social network environment	Online social network	honesty, reputation, competency, credibility, confidence, reliability, belief, faith	Quantitative method on survey questionnaire data	This model can address trust issues on social networking sites through personalized trust features
Quinn et al [27]	Multifaceted trust model	Internet	honesty, reputation, competency, credibility, confidence, reliability, belief, faith	Quantitative method on survey questionnaire data	A multifaceted model is personalizable and specializable; provides accuracy of trust recommendation
Montaque et al [61]	Model of patient and provider trust in medical technology	Health care	Communication, compassion, privacy, competence, confidentiality, dependability, reliability	Qualitative method with a grounded theory approach	The interaction between the provider and technology influences patient trust in technology
Zahedi and Song [65]	Dynamic model of trust	Health care	ability, benevolence, integrity	Quantitative method on a laboratory experiment	Trust beliefs change depending on web consumers with more experience in health infomediaries.
Corritore et al [66]	Model of online trust	Health care	Credibility, risk, ease of use	Quantitative method on the instrument of a 34-item Likert-scale	To lead the development of the health care website on trust; produce a valid instrument to measure online trust on the health care website; produce a model of online trust for health care websites
Lee and Turban [67]	A trust model for consumer internet shopping	Internet	Ability, benevolence, integrity, trust propensity	Quantitative method on survey questionnaire data	Merchant integrity is a major positive determinant of consumer trust and its effect.
Lewicki et al [64]	Models of interpersonal trust development	Psychology	Ability, benevolence, integrity	Qualitative method on the grounded theory approach	A behavioral and physiological approach theory
Dibben et al [62]	A model of trust development in the patient-physician relationship	Health care	Dispositional trust, learnt trust, situational trust	Qualitative method	“This model is able to identify and map trust levels and thresholds of cooperative behavior and modify the behavior on the interaction between physician and patient.”

Based on analysis of the 11 filtered articles, we decided to adopt the multifaceted trust model introduced by Quinn et al [27]. The reason for selecting the multifaceted trust model (Table 4) is that this model can provide a subjective view of individual trust for each user. This model is also a user-centric model that can personalize trust features such as comments and photos on social media [30]. In particular, trust can protect the physician’s reputation before the patient makes any decision and allows the user to choose a credible physician [44]. According to Singh and Chin [36], trust is a significant factor to attract a user to use the site for recommendation to others based on a rating feature. That is, patients can consider a physician’s credibility for consultations, and physicians can consider the patient’s honesty in providing information about their health status [36].

### Evaluation of the Multifaceted Trustworthiness of the Breast Self-Examination System

#### Correlation Analysis Among Trustworthiness Indicators

The Pearson correlation coefficient (r) was used to evaluate the correlations among various trustworthiness indicators in the breast self-examination system based on the following formula:


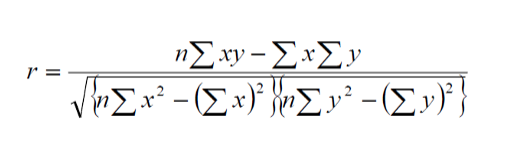


The Pearson correlation coefficient represents a relationship (*r*) between the independent variable (*x*) and the dependent variable (*y*) based on a numerical variable between –1 and 1, where 0 indicates no correlation, 1 indicates a complete positive correlation, and –1 indicates a complete negative correlation. A correlation coefficient of 0.7 and above indicates a significant and positive relationship between *x* and *y*; that is, when variable *x* increases, variable *y* will also increase. Similarly, if the correlation value is negative, if *x* increases, then *y* also decreases [68]. We conducted a correlation analysis from 77 samples collected from the MySQL database, which included ratings of patients for a doctor in a chat room, ratings of patients for several doctors in the chat room, and ratings of patients for a doctor in the forum. After a participant chatted with a doctor in a chat room, the participant could rate the doctor based on 8 trustworthiness indicators, and then was required to edit the rating after a second consultation. The data were exported from MySQL to a Microsoft Office Excel spreadsheet, and Pearson correlation analysis was performed using SPSS v.24 (IBM) [69-71]. The Cronbach α value was .92, which means that the data are reliable (>.80) [71,72]. The results of the correlation analysis are summarized in Table 5.

**Table 5:** Correlation coefficients of each trustworthiness indicator and trust value.

Trustworthiness indicators	Correlation coefficient
honesty	0.91
reputation	0.72
competency	0.73
reliability	0.73
credibility	0.85
belief	0.75
confidence	0.79
faith	0.79

A strong positive correlation (>0.70) was found for all trustworthiness indicators and trust value [68], indicating that trustworthiness indicators would predict a tendency to change the trust value; the higher the values of the correlation coefficient, the stronger the relationship between the trustworthiness indicator and trust value. The highest trustworthiness indicator with a strong positive correlation was honesty, followed by credibility, confidence, and faith. Reputation had the lowest correlation value among the trustworthiness indicators (Table 5). The correlations among trustworthiness indicators were significant (*P*<.001). Overall, these results suggest that honesty has the highest rank with respect to trustworthiness, which means that the most important aspect in the interaction process between a doctor and patient is honest communication. This is followed by credibility and faith as the second most important factors, reflecting the need of patients to have a doctor with good credibility.

#### Patient-Physician Relationship

The patient-physician interaction was evaluated to measure the trust level of a patient toward a physician’s behavior [55]. This analysis was based on different views such as the interaction in different time frames, patient interactions with several physicians, and patient feedback on physicians’ articles in the forum.

##### Patient and Physician Interaction in Different Time Frames

The patients’ ratings of a physician in the chat room over multiple interactions are summarized in Table 6. For example, Patient X5 had a consultation with physician Y5 on different occasions. The trust value in the first week was 8.63 and was 10.00 in the second week. Similarly, for patient X13 and physician Y6, the trust value in the first week was 8.13, which increased to 8.50 and 9.25 in the third and fourth week, respectively. This interaction shows that the trust level increased from medium to high, which means that the trust value and trust level of the patient for a particular physician will generally increase after several interactions. Indeed, the time effect in the interaction analysis based on comparing the trust value between the first and second week was statistically significant (*P*=.03).

**Table 6:** Interaction between a patient and a doctor over different time frames.

Patient	Doctor	Meeting time	Trustworthiness indicators (ratings)															Trust value		Trust level	
			honesty		reputation	competency		reliability		credibility		belief		confidence		faith					
X4	Y3	Week 1	10		10	9		10		10		8		9		10		9.50		High	
X4	Y3	Week 2	10		10	10		10		9		9		10		10		9.75		High	
X5	Y5	Week 1	10		9	8		8		8		9		9		8		8.63		Medium	
X5	Y5	Week 2	10		10	10		10		10		10		10		10		10.00		High	
X7	Y10	Week 1	8		7	8		9		9		9		7		8		8.13		Medium	
X7	Y10	Week 2	9		8	9		9		9		9		8		9		8.75		Medium	
X7	Y10	Week 3	10		9	9		9		9		9		9		8		9.00		High	
X11	Y12	Week 1	7		9	9		8		8		9		10		9		8.63		Medium	
X11	Y12	Week 2	8		10	7		9		10		9		9		9		8.88		Medium	
X12	Y3	Week 1	9		10	10		9		10		10		10		9		9.63		High	
X12	Y3	Week 2	10		10	10		10		10		10		10		10		10.00		High	
X13	Y6	Week 1	7		9	8		7		9		8		8		9		8.13		Medium	
X13	Y6	Week 2	8		9	9		7		9		9		9		8		8.50		Medium	
X13	Y6	Week 3	10		10	9		9		10		8		10		8		9.25		High	
Total Average				9.00	9.29		8.93		8.86		9.29		9.00		9.14		8.93		9.06		High

As shown in Table 6, the trust value increased when each patient had several consultations with a physician on different occasions. The trust value was taken from each patient’s feedback rating, including patients X4, X5, X7, X11, X12, and X13, who provided useful input on trustworthiness regarding their physician after several consultations.

##### One Patient With Many Physician Interactions

Data interaction in the chat room demonstrated that several patients interacted with more than one physician. Sixteen patients requested communication with several physicians, and only 11 physicians responded to these requests and had an excellent interaction with patients. Patients prefer to chat with physicians who have been rated by another patient. However, not all physicians were receptive to accepting a patient request due to their limited time. Table 7 demonstrates the varying trust values between physicians during interactions with the same patient. The trust value is given by the patients that provided their subjective views when rating a physician after consultation. Based on the total average of trustworthiness indicators, honesty (9.39) emerged as the most important indicator, followed by credibility (9.28) and faith (9.28).

**Table 7:** Patient ratings of several physicians in a chat room.

Patient	Doctor	Trustworthiness indicators								Trust value		Trust level	
		honesty	reputation	competency	reliability	credibility	belief	confidence	faith				
X1	Y1	9	8	9	9	8	9	9	9		8.75		Medium
X1	Y2	9	10	9	10	10	10	10	10		9.75		High
X2	Y3	10	10	10	10	10	10	10	10		10.00		High
X2	Y4	10	8	10	10	10	10	9	10		9.63		High
X3	Y3	10	8	10	10	10	10	10	10		9.75		High
X3	Y6	9	10	9	10	10	10	10	10		9.75		High
X3	Y5	9	10	9	10	10	10	10	10		9.75		High
X4	Y3	10	10	9	10	10	8	9	10		9.50		High
X4	Y5	10	10	10	9	8	9	9	8		9.13		High
X5	Y5	10	9	8	8	8	9	9	8		8.63		Medium
X5	Y6	8	9	8	8	9	8	7	8		8.13		Medium
X6	Y7	9	9	9	9	9	9	9	9		9.00		High
X6	Y8	9	8	9	8	8	9	9	9		8.63		Medium
X7	Y9	9	9	9	8	9	9	10	9		9.00		High
X7	Y2	10	9	10	9	9	9	9	10		9.38		High
X7	Y10	8	7	8	9	9	9	7	8		8.13		Medium
X8	Y11	10	8	9	8	10	8	8	9		8.75		Medium
X8	Y2	10	8	10	8	10	8	9	10		9.13		High
Total Average		9.39	8.89	9.17	9.06	9.28	9.11	9.06	9.28		9.16		High

##### Rating of Physician-Posted Articles by Patients in the Forum

In the forum, 9 physicians participated in posting information (articles) related to breast cancer, and 13 patients rated the articles. As shown in Table 8, the trustworthiness ratings on the forum revealed the highest trust value for physician Y15, followed by physicians Y7 and Y12. This means that physician Y15 posted the most highly trusted articles even though physician Y12’s article was read by more patients.

**Table 8:** Trust value matrix on patient (X) ratings of articles posted by physicians (Y).

Patient	Y5	Y7	Y12	Y13	Y14	Y15	Y16	Y17	Y18
X1	—^a^	—	—	—	—	—	—	—	8.29
X2	—	—	—	—	—	—	—	8.29	8.71
X3	8.43	—	8.43	—	8.00	—	—	—	—
X4	8.43	—	8.14	—	—	—	—	—	—
X5	—	8.57	—	—	8.71	—	—	—	—
X6	—	—	9.57	—	9.00	9.29	—	—	—
X7	8.57	—	8.57	—	—	—	—	—	—
X9	—	—	—	—	—	8.71	8.57	8.71	—
X10	—	—	—	—	—	9.00	8.86	8.57	—
X11	—	—	—	8.00	—	—	—	—	—
X12	—	—	—	—	—	—	—	—	8.14
X14	—	9.00	—	—	—	—	—	—	—
Total (trust level)	8.48 (medium)	8.79 (medium)	8.68 (medium)	8.00 (medium)	8.57 (medium)	9.00 (high)	8.72 (medium)	8.52 (medium)	8.38 (medium)

^a^—: data not applicable, given no rating for that physician’s article.

Table 9 shows the trust values for each physician rated by several patients. Patients rated a physician based on their personal views on the articles posted in the forum by the physicians. For example, physician Y14 was rated by three different patients (X3=8.00, X5=8.71, and X6=9.00), indicating a very high trust level by patient X6. This means that patient X6 considered the article posted by physician Y14 to be of more benefit compared with the views of patients X3 and X5. In this case, honesty (8.86) was the most important indicator, followed by belief (8.81) and confidence (8.81).

**Table 9:** Patient ratings of articles posted by physicians on the forum.

Doctor	Patient	Trustworthiness indicators								Trust value		Trust level	
		honesty	reputation	competency	reliability	credibility	belief	confidence	faith				
Y14	X6	9	9	9	9	9	8	10	8		9.00		High
Y14	X5	9	8	9	9	8	9	9	8		8.71		Medium
Y14	X3	8	9	8	8	8	7	8	9		8.00		Medium
Y15	X6	10	9	9	9	8	10	10	9		9.29		High
Y15	X9	9	8	9	9	8	9	9	8		8.71		Medium
Y15	X10	10	9	9	8	8	10	9	8		9.00		High
Y12	X6	10	9	10	9	9	10	10	9		9.57		High
Y12	X7	9	9	9	9	8	8	8	9		8.57		Medium
Y12	X3	9	9	9	8	7	8	9	8		8.43		Medium
Y12	X4	7	8	8	8	9	8	9	9		8.14		Medium
Y16	X9	8	9	9	9	8	8	9	9		8.57		Medium
Y16	X10	9	8	9	8	8	10	10	7		8.86		Medium
Y17	X9	9	9	8	9	9	8	9	8		8.71		Medium
Y17	X2	8	8	8	8	8	9	9	9		8.29		Medium
Y17	X10	10	9	8	9	7	9	8	8		8.57		Medium
Y5	X7	9	8	8	8	9	9	9	9		8.57		Medium
Y5	X3	8	9	8	9	9	9	7	9		8.43		Medium
Y5	X4	9	9	8	8	9	8	8	7		8.43		Medium
Y18	X1	8	8	9	8	9	8	8	7		8.29		Medium
Y18	X2	9	9	9	8	8	9	9	9		8.71		Medium
Y18	X12	9	8	8	8	9	8	7	7		8.14		Medium
Y7	X5	9	9	8	8	8	9	9	9		8.57		Medium
Y7	X14	10	9	9	9	9	9	8	9		9.00		High
Y13	X11	8	8	8	7	8	10	7	9		8.00		Medium
Total Average		8.88	8.63	8.58	8.42	8.33	8.75	8.67	8.38		8.61		Medium

## Discussion

Eight indicators of trustworthiness taken from the multifaceted trust model showed significant positive correlations with trust value, including honesty, credibility, confidence, faith, belief, competency, reliability, and reputation. The following nine features were considered to be important in the design of the breast self-examination system: user account, calendar, self-exam wizard, history, chat room, location, knowledge, video tutorial, and forum. The trust level of a patient for a particular physician was found to increase after several interactions, and the patient can choose the right physician by considering other patients’ recommendations based on the physician’s trust level.

There are 32 participants registered in the breast self-examination system. Registration is achieved through a user account with approval validation sent by the system to the user’s email. The security model of the user account is MD5. Users set their cycle period via the calendar and follow the self-exam wizard by recording their activity in the history feature. If a patient identifies changes on the surface of their breast during a self-exam, they can take a photo of the breast and enter it into the system, which can be annotated as a “sensitive” picture [36,44]. A picture that is annotated as sensitive is then assigned a weight for category (Wc), which means that the picture will require the patient’s informed consent before sharing with the physician. The chat room is a convenient space for interaction and communication between a patient and physician. By default, the patients deidentify themselves by showing only their patient ID number to the physician. During the interaction and at the physician’s request, the patient shares their history as their medical record. This sharing was identified as a weight for action (Wa). The Wa will lead the patient to share based on the request from the physician. On the other side, physicians are able to post an article to the forum*,* which is identified as a weight for the post (Wp). The patients looked at several articles posted by the physician in the forum and provided feedback through rating the physician. Each share, post, and category is a confidential activity carried out by the patient and physician [36,44].

The correlation analysis of trustworthiness factors on the breast self-examination system demonstrated that honesty has the highest ranking for trustworthiness overall. This reflects that the interaction process between physicians and patients requires honest communication through honest information from the patient so that the physician can provide the correct treatment. Honest advice from the physician will create trust on the patient’s part, and as a result, the patient will follow the physician’s advice. This was followed by credibility as the second most important feature due to the patient requiring a credible doctor [27].

The analysis of patient-physician interaction over different time frames revealed that patient trust will grow when several interactions occur between a patient and physician. The patient’s understanding of the physician regarding their reputation and credibility is the first preference. Some feedback from the patients included feeling comfortable talking with physicians based on a recommendation by another patient through seeing the physician trust value. This feedback proves that trust is indeed transitive. The interaction of one patient with several physicians reflects the personal views of the patient about a particular physician based on their convenient communication in the chat room [30].

Patient feedback in the forum related to articles posted by a physician was based on the valuable information received by the patient, indicating that patients have their own views for accessing the useful information provided in each article posted by a physician. The most trusted article was measured by the weight of trust value. Overall, we found that patients’ subjective views in taking the information from each posted article on breast cancer benefited the patients based on their own experience and situation (ie, context-dependent effect) [30].

Overall, this study reveals the strong ability of the multifaceted trust model to provide a more trustworthy system, ethical interactions between patients and physicians, and patient control of data. This analysis proves the trust characteristic of social media through interactions between patients and physicians in the breast self-examination system [63,73]. Ultimately, the implementation of multifaceted trust enables patients to make the right choice of a physician by considering other patients’ recommendations based on the physician’s trust level.

In conclusion, multifaceted trustworthiness indicators have a significant impact on the breast self-examination system. These indicators provide a trustworthy system and ethical interaction between a patient and physician as assessed through the trust value and trust level. Based on the trust value and trust level of physicians, a new patient can obtain a consultation by referring to the most highly preferred physician. In addition, the patient’s trust level to a particular physician will increase after several interactions. The correlation analysis also showed that the most preferred trustworthiness indicator was honesty. With more interactions that occur based on weekly meetings, more trust will grow between the patient and physician. This trust will automatically increase the reputation and credibility of the physician.

Multifaceted trustworthiness could be explored in more areas of relevance in the health care system. Several actors in various health care systems should consider adding and reviewing the process of interactions, such as those occurring among the health care provider, patient, physician, system provider, and health care supplier.

## Appendix

Multimedia Appendix 1 CONSORT-EHEALTH checklist (V 1.6.1).

## Abbreviations

ARV: average rating value
TISoN: trust inference for social networks
Wa: weight of action
Wc: weight of category
Wp: weight of post
